# The murine Microenvironment Cell Population counter method to estimate abundance of tissue-infiltrating immune and stromal cell populations in murine samples using gene expression

**DOI:** 10.1186/s13073-020-00783-w

**Published:** 2020-10-06

**Authors:** Florent Petitprez, Sacha Levy, Cheng-Ming Sun, Maxime Meylan, Christophe Linhard, Etienne Becht, Nabila Elarouci, David Tavel, Lubka T. Roumenina, Mira Ayadi, Catherine Sautès-Fridman, Wolf H. Fridman, Aurélien de Reyniès

**Affiliations:** 1 Centre de Recherche des Cordeliers, INSERM, Sorbonne Université, Université de Paris, Team Inflammation, Complement and Cancer, F-75006 Paris, France; 2 Programme Cartes d’Identité des Tumeurs, Ligue Nationale contre le Cancer, F-75013 Paris, France; 3 Present address: MRC Centre for Reproductive Health, The University of Edinburgh, The Queen’s Medical Research Institute, Edinburgh, UK; 4 Fred Hutchinson Cancer Research Center, Seattle, WA USA

**Keywords:** Immune composition, Heterogeneous tissue, Tumor microenvironment, Immune checkpoint blockade, Alzheimer’s disease

## Abstract

Quantifying tissue-infiltrating immune and stromal cells provides clinically relevant information for various diseases. While numerous methods can quantify immune or stromal cells in human tissue samples from transcriptomic data, few are available for mouse studies. We introduce murine Microenvironment Cell Population counter (mMCP-counter), a method based on highly specific transcriptomic markers that accurately quantify 16 immune and stromal murine cell populations. We validated mMCP-counter with flow cytometry data and showed that mMCP-counter outperforms existing methods. We showed that mMCP-counter scores are predictive of response to immune checkpoint blockade in cancer mouse models and identify early immune impacts of Alzheimer’s disease.

## Background

For a large number of diseases, such as inflammatory diseases or cancer, it is often crucial to accurately determine the cellular composition of the tissue where the pathology develops, in terms of immune and stromal cell populations. An array of methods are available to obtain these data from human samples, either by immunochemistry or cytometry, or computationally from transcriptomics data [1].

The analysis of the immune and stromal composition of tissues is particularly critical in cancer studies. Indeed, tumors are highly heterogeneous tissues which are infiltrated by a variety of immune and stromal cells [2]. It was shown that immune cell densities were associated with prognosis [3]. For instance, CD8^+^ T cells density correlates with prolonged patient survival in most cancers, whereas M2-polarized macrophages are generally associated with a poor prognosis [3].

The transcriptome of a bulk tissue sample yields the averaged expression of genes across all the cells present in the sample. As some genes are uniquely expressed in some specific cell populations, their expression can be used to determine the abundance of the underlying cell populations. Using this property, we have previously reported on MCP-counter, a method designed to quantify the immune infiltrate of heterogeneous human tissues [4], currently one of the best performing methods for this purpose [5].

While murine models are widely used to decipher the pathophysiological mechanisms of various diseases, including inflammatory diseases and cancer, the computational methods currently available to measure the immune and stromal composition of murine tissues are few and limited, as compared to what is available for human samples [6].

**Fig. 1:**
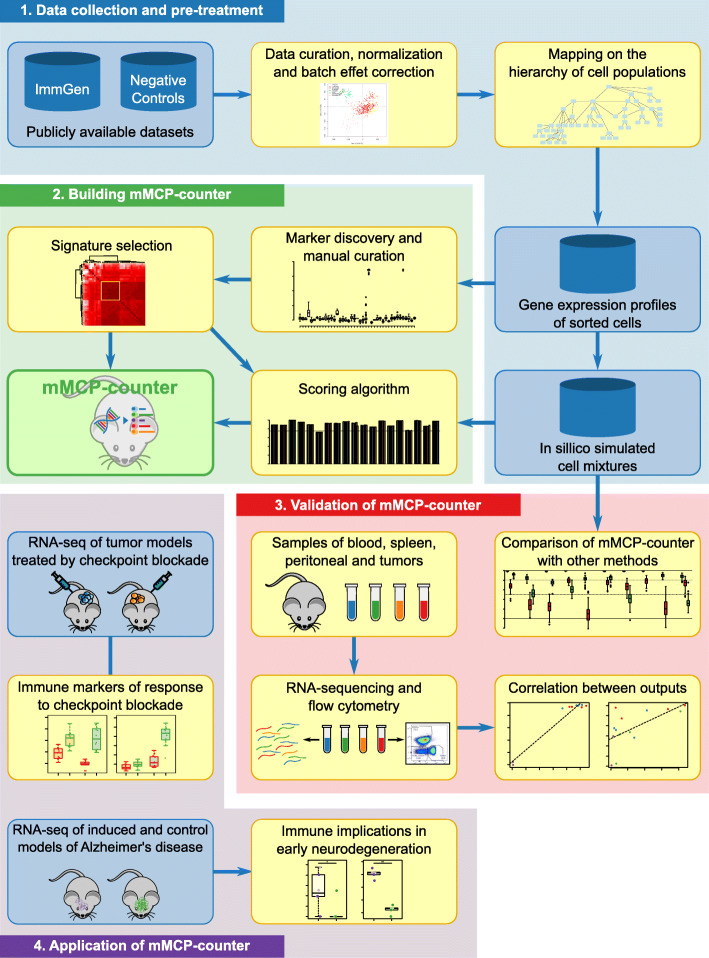
Workflow for the development, validation, and application of mMCP-counter. This figure depicts (1) the data acquisition, pre-processing, and normalization, as well as the mapping the cell population hierarchy; (2) the building of the methods by research and curation of cell-type-specific gene signatures and optimal scoring algorithm; (3) the validation of mMCP-counter by comparison to previously published methods on simulated mixtures and by comparison to immune composition inferred by flow-cytometry; and (4) the illustration of mMCP-counter to two datasets including mouse models of kidney cancer and mesothelioma treated by immune checkpoint blockade, and murine models of early neurodegeneration in Alzheimer’s disease

Here, we introduce murine Microenvironment Cell Populations counter (mMCP-counter), the adaptation of the MCP-counter method to murine samples (Fig. 1), which was made possible thanks to the release of large datasets of microarray-measured gene expression of murine sorted immune populations by the Immunological Genome Project (ImmGen) [7, 8]. mMCP-counter can be accessed as an R package (https://github.com/cit-bioinfo/mMCP-counter) [9]. It takes a gene expression profiles matrix as input and returns the abundance of RNA originating from 16 defined cell populations present in the heterogeneous sample.

We compared the performance of mMCP-counter with other previously published methods on simulated mixtures generated using two datasets that are independent from the discovery ImmGen microarrays datasets, and we validated our approach on samples from peripheral blood, peritoneum, spleen, and several grafted tumors that were analyzed by both RNA-sequencing (RNA-Seq) and flow cytometry (Fig. 1). Finally, we analyzed how mMCP-counter can be used, in murine models of mesothelioma and kidney cancer, to analyze the TME differences between responders and non-responders to immune checkpoint blockade, a crucial and emerging therapy for many cancer types, and in a murine model of early neurodegeneration from Alzheimer’s disease, to identify immune and stromal cell populations in hippocampal transcriptomes.

## Methods

### Public data accession, curation, and normalization

Data included in the discovery dataset included the two micro-array releases of ImmGen (Gene Expression Omnibus (GEO) accession numbers GSE15907 and GSE37448), and parts of several datasets for: epithelial cells (GSE27456 and GSE74317), breast cancer (GSE25525, GSE54626, and GSE78698), hypothalamic cell line (GSE61402), melanoma B16F10 cells (GSE84155), pancreatic ductal adenocarcinoma (GSE48643), myoblasts (GSE26764), and hepatocytes (GSE18614). Raw CEL files were used and data was normalized through frozen robust multi-array analysis [10] with the R package fRMA. Batch effect was correcting using ComBat [11] from the R package sva. Consistency within the data was verified using principal components analysis with the R package FactoMineR [12] and outliers were discarded.

Application data were downloaded from GEO (accession numbers GSE93017 and GSE117358). For GSE117358, data was normalized at the 75th percentile of gene expression.

### Signatures discovery

For signature discovery, only populations for which all subsets were present in the dataset and that have appropriate negative samples were taken into account. All probes were screened for (log2) fold-change (FC), specific fold change (sFC), and area under the ROC curve (AUC). FC and sFC are defined as follows:

$$ FC=X-\overline{X} $$

$$ \mathrm{sFC}=\frac{X-{\overline{X}}_{\mathrm{min}}}{{\overline{X}}_{\mathrm{max}}-{\overline{X}}_{\mathrm{min}}} $$

where *X* is the centroid (i.e., the mean over all samples) for the population of interest, $\overline{X}$ the centroid of all other samples, and ${\overline{X}}_{\mathrm{max}}$ and ${\overline{X}}_{\mathrm{min}}$ denote, respectively, the maximum and the minimum of cell-type-specific centroids for population different from the population of interest.

Signatures were built using the following cut-offs: FC > 2.1, sFC > 2.1, and AUC > 0.97. After this automated screening, all retained probed were manually curated to verify the accuracy of the selection. All signatures that contained more than 8 putative transcriptomic markers underwent an additional selection process. A sub-signature with strong inter-marker correlation was kept following hierarchical clustering of the whole signature transcriptomic markers. The hierarchical clustering was made using R, with Euclidian metric and Ward’s linkage criterion.

### In silico mixtures preparation

The in silico simulated mixtures were computed as follows: firstly, weights for all included populations were chosen randomly. Pure transcriptomic profiles for all populations were computed with the expression of all genes being the mean expression over all the corresponding samples in the Haemopedia and ImmGen ULI datasets. Finally, the mixture transcriptome was computed as follows:

$$ T=P\times C $$

where *T* is the transcriptomic matrix with genes in lines and samples (mixtures) in columns, *P* is the pure profiles matrix with genes in lines and cell populations in columns, and *C* is the mixture composition matrix, with populations in lines and samples (mixtures) in column, the sum of each column being equal to 1.

To evaluate the various scoring algorithms, 24 mixtures were simulated with random proportions of each cell population. For the comparisons between mMCP-counter and other methods, 50 sets of mixtures were generated from the Haemopedia data (accessed as TPM-normalized data from www.haemosphere.org and log2-transformed) and ImmGen ULI data (accessed as raw counts from Gene Expression Omnibus (accession code GSE109125), normalized at the 75th percentile and log2-transformed). For the Haemopedia data, the random proportions for 50 mixtures were simulated using a Dirichlet distribution with shape parameters 2.8 (CD8 T cells), 2.2 (other T cells), 1.8 (B cells), 0.5 (monocytes), 1.7 (macrophages), 0.2 (mast cells), 0.5 (eosinophils), 0.3 (neutrophils). For the ImmGen ULI dataset mixtures, the shape parameters were set to 2.8 (CD8 T cells), 0.2 (gamma-delta T cells), 2 (other T cells), 0.2 (NK cells), 0.8 (memory B cells), 0.8 (other B cells), 0.5 (monocytes), 2 (macrophages), 0.2 (mast cells), 0.2 (eosinophils), and 0.3 (neutrophils).

### Comparison with other published methods

mMCP-counter, DCQ, and ImmuCC were run independently on each of the 50 sets of 50 mixtures defined above, aggregated by gene. The ImmuCC algorithm and signature matrix was accessed on GitHub (https://github.com/chenziyi/ImmuCC) and ran on the mixtures locally. DCQ was run using the dcq function from the ComICS R package. All methods were run using default parameters. When the granularity of cell populations differed between mMCP-counter and ImmuCC or DCQ, to allow for comparisons, we forced a similar granularity, summing the scores of subpopulations corresponding to a larger population. For ImmuCC, populations “T Cells CD8 Actived,” “T Cells CD8 Naive,” “T Cells CD8 Memory,” “T Cells CD4 Memory,” “T Cells CD4 Naive,” “T Cells CD4 Follicular,” and “GammaDelta T Cells” were summed as “T cells”; “T Cells CD8 Actived,” “T Cells CD8 Naive,” and “T Cells CD8 Memory” were summed as “CD8 T cells”; “NK Resting” and “NK.Actived” were summed as “NK cells”; “B Cells Memory,” “B Cells Naive,” and “Plasma Cells” were summed as “B derived”; “M0 Macrophage,” “M1 Macrophage,” “M2 Macrophage,” and “Monocyte” were summed as “Monocytes / macrophages.” For DCQ, all populations starting with “T.” or “TGD.” were summed as “T cells”; all populations starting with “T.8” were summed as “CD8 T cells”; all populations starting with “NK.” were summed as “NK cells”; all populations starting with “B.” were summed as “B derived”; all populations starting with “MO.” or “MF.” were summed as “Monocytes/macrophages”; and all populations starting with “GN.” were summed as “neutrophils.”

### Spillover analysis

For spillover, mean profiles for CD8^+^ T cells, B-derived cells, monocytes/macrophages, neutrophils, eosinophils, and mast cells from the Haemopedia dataset, and the above populations plus NK cells from the ImmGen ULI dataset were computed. All three methods were applied on these pure profiles, and the results were aggregated to higher-level cell populations as in the above paragraph. The noise ratio was computed with the following formula:

$$ \mathrm{noise}\ \mathrm{ratio}=\frac{\mathrm{noise}}{\mathrm{noise}+\mathrm{signal}} $$

where noise is the sum of all off-targets scores (i.e., scores for all other populations than what was included in each case) and signal is the sum of target scores (i.e., the scores for the correct populations). For DCQ, we substracted the minimum value to all scores in order to only have positive values.

### Animal experiment

Eight- to 10-week-old female C57/BL6 mice were purchased from Charles River Laboratories. The use of animals followed the institutional guidelines and the recommendations for the care and use of laboratory animals with approvals APAFIS#34\0-2016052518485390v2 and #9853-2017050211531651v5 by the French Ministry of Agriculture. Mice were sacrificed and spleens were surgically removed and were pressed through a 70-μm cell strainer (BD Falcon) for single-cell suspension preparation. Blood was obtained with a cardiac puncture or from the submandibular vein. Peritoneal cells were obtained by washing the peritoneal cavity with 3–4 ml of PBS twice. Red blood cells were lysed by ACK lysing buffer and cells were then washed with PBS with 2% of fetal bovine serum (FBS). All cells were resuspended in ice-cold PBS with 2% FBS for FACs staining.

TC-1, tumor cells derived from mouse lung epithelial cells and transformed by human papillomavirus [13], were cultured in vitro. Cells were washed with PBS and 4 × 10^5^ cells were inoculated subcutaneously in the right flank with 200 μl PBS. Twenty-six days later, tumors were surgically removed and single-cell suspension is prepared for FACs analysis.

### Flow cytometry

For flow cytometry, cells were stained with the following monoclonal antibodies: PE-conjugated anti-CD4, Fitc-conjugated anti-CD8, BV786-conjugated anti-CD11c, PE-Cy7-conjugated anti-CD45, BV605-conjugated anti-NK-1.1 (all from BD Biosciences), eFluor 450-conjugated anti-CD11b, Alexa Fluor 700-conjugated anti-CD19, APC-eFluor 780-conjugated anti-CD19, PerCP-eFluor 710-conjugated anti-CD49b, PE-CF594-conjugated anti-Siglec-F, (all from eBioscience), Brilliant Violet 785-conjugated anti-CD11b, APC/Fire 750-conjugated anti-TCR-β, Pacific Blue-conjugated anti-GL7, Fitc-conjugated anti-FcεRIa, Alexa Fluor 700-conjugated anti-F4/80, Brilliant Violet 605-conjugated anti-Ly-6C, and Brilliant Violet 650-conjugated anti-Ly-6G (all from BioLegend). Cells were stained for 30 min in the dark at 4 °C and were washed with PBS with 2% of FBS. For Foxp3 staining, cells were fixed and permeabilized with eBioscience Foxp3/Transcription Factor Staining Buffer Set according to the manufacturer’s protocol (eBioscience). All stainings were done with Fc block (BD Biosciences). Cells were then analyzed on a BD LSRFortessa cell analyzer (BD Bioscience). Flow cytometry data analysis was performed using Flowjo analysis platform (FlowJo, LLC).

Living cells were identified by LIVE/DEAD Fixable aqua dead cell stain kit (ThermoFisher Scientific) and singlet cells were gated before further analysis. T cells are identified as CD19^−^B220^−^CD11b^−^NK1.1^−^TCRβ^+^ cells, CD8^+^ T cells are CD19^−^B220^−^CD11b^−^NK1.1^−^TCRβ^+^CD8^+^ cells, T_reg_ cells are CD19^−^B220^−^TCRβ^+^CD4^+^CD25^+^Foxp3^+^ cells, NK cells are CD19^−^TCRβ^−^NK1.1^+^ cells, B-derived cells are CD19^+^B220^+^TCRβ^−^ cells, memory B cells are CD19^+^B220^+^CD38^+^CD80^+^IgD^lo^ cells, neutrophils are CD19^−^TCRβ^−^CD11c^−^CD11b^+^Ly6G^+^ cells, eosinophils are CD19^−^TCRβ^−^CD11c^−^CD11b^lo^Ly6G^−^SiglecF^+^ cells, basophils are CD19^−^TCRβ^−^CD11c^−^CD11b^+^Ly6G^−^CD117^−^FcεRIa^+^CD49d^+^ cells, mast cells are CD19^−^TCRβ^−^CD11b^−^FcεRIa^+^CD117^+^ peritoneal cells, monocytes are CD19^−^TCRβ^−^CD11c^−^F4/80^−^CD11b^+^CD115^+^ cells, and macrophages are CD19^−^TCRβ^−^F4/80^+^CD11b^+^ peritoneal cells.

### RNA preparation

Splenocytes, peripheral blood cells, peritoneal cells, and tumor cells were washed with PBS and were counted. Cells were centrifuged at 400*g* for 5 min and supernatant was removed. Cells pellet (< 3 × 10^6^ cells) was resuspend in 350 μl of RLT buffer (Qiagen). RNA was extracted with RNeasy Mini kit (Qiagen) according to the manufacturer’s protocol.

### RNA sequencing

mRNA library preparation was realized following the manufacturer’s recommendations (KAPA mRNA HyperPrep ROCHE). Library purity/integrity were assessed using an Agilent 2200 Tapestation (Agilent Technologies, Waldbrunn, Germany). Final 7 samples pooled library prep were sequenced on Nextseq 500 ILLUMINA with MidOutPut cartridge (2 × 130 million of 75 bases reads), corresponding to 2 × 18 million of reads per sample after demultiplexing.

### RNA-seq data pre-processing and normalization

Raw RNA-seq reads were aligned on the GCRm38 *Mus musculus* genome using STAR pipeline. Feature count was performed using the Rsubread R package and normalized at the 75th percentile.

### Analysis of single-cell RNA-seq data

The analysis of the single-cell RNA-seq data from the *Tabula Muris* consortium [14] was accessed on the Single Cell Expression Atlas [15] from EMBL-EBI at https://www.ebi.ac.uk/gxa/sc/home on March 4, 2020, and analyzed online on this platform.

### Statistical analysis

All statistical analyses were made using R 3.6.2 with packages gtools, ComICS, circlize [16], and ComplexHeatmap [17]. Pearson’s correlation was used to compare two quantitative variables. The comparisons between mMCP-counter and other methods (Fig. 4) were assessed using *t* tests. For other comparisons between a quantitative variable and a 2-level qualitative variable, we used Mann-Whitney tests. For 3 or more levels, we used Kruskal-Wallis with post hoc Dunn test for pairwise comparisons with Benjamini-Hochberg correction. Associations between two quantitative variables were assessed using Pearson’s correlation and its correlation test.

## Results

### Prior hierarchization of cell populations

mMCP-counter relies on the identification of specific transcriptomic markers for each analyzed population. We define transcriptomic markers as having a “high” expression in a given cell population, including all its subpopulations, and “zero” expression (meaning either zero or not differentiable from the detection threshold, depending on the technologies) in any other cell population. Their detection is based on three criteria (see *signature discovery* thereafter). This approach requires to represent a priori both the cell categories and their inclusion relationships, as comprehensively as possible. To do so, we completed the hematopoietic tree provided by ImmGen [18], using a survey of the literature [19, 20]. The resulting hierarchy of cell populations is presented in Additional file 1: Fig. S1a.

### Constitution of a training series

To obtain enough transcriptomic samples mapping to each of the nodes (cell categories) of the prior hierarchical model, we collected transcriptomic profiles of sorted cell populations from the ImmGen Microarray datasets. To include non-immune non-stromal negative controls, parts of additional datasets were also added to our training data, including those from epithelial cell lines, hypothalamic cell lines, melanoma, pancreatic ductal adenocarcinoma, and breast cancer cell lines, myoblasts, and hepatocytes. Curation and normalization of data are explained in the “Methods” section.

### Signatures discovery

**Fig. 2:**
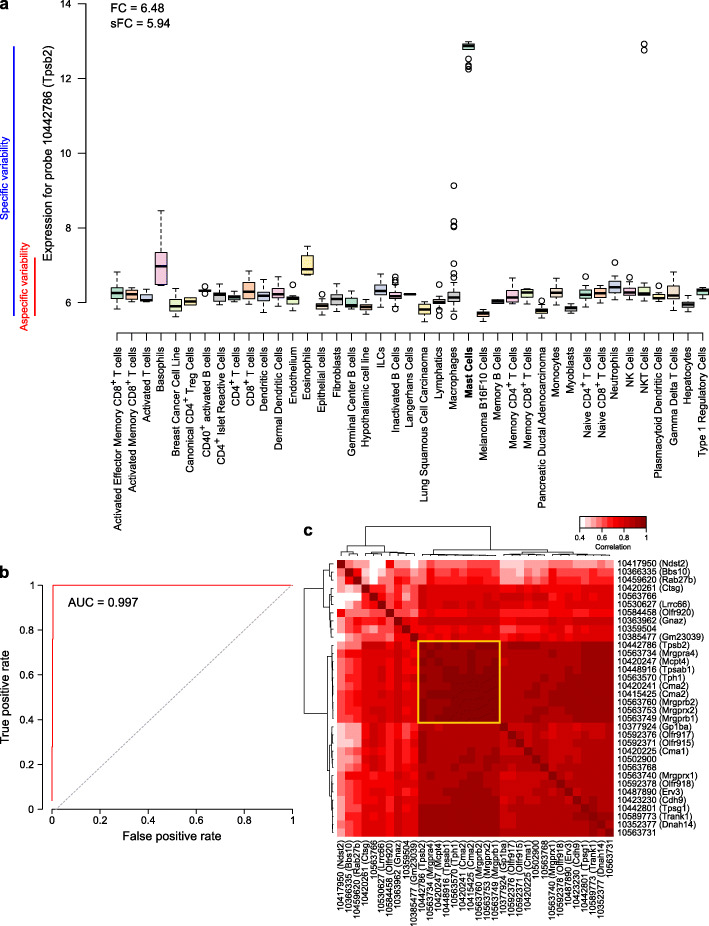
Identification of cell-type-specific gene signatures: example of mast cells. **a** Expression of a transcriptomic marker (probe 10442786) of mast cells in various cell types, with the representation of the fold-change and the specific fold-change. **b** Receiver operating characteristic (ROC) curve for the same marker as in **a**. **c** Correlation heatmap of all found transcriptomic markers for mast cells. The yellow square indicates the restricted signature that was chosen for the method

Only categories that were fully represented, i.e., of which all subcategories were included in the dataset, were considered for signature discovery. For each of the 55 remaining populations (Additional file 1: Fig. S1b), all available transcripts (probes) were screened for (log2) fold-change (FC), specific fold change (sFC), and area under the ROC curve (AUC) (see the “Methods” section). Features were considered as transcriptomic markers for a given population if they respected 3 criteria: FC > 2.1, sFC > 2.1, and AUC > 0.97. Figure 2 a and b illustrate an example with probe 10442786 (*Tpsb2*), which qualified as a transcriptomic marker for mast cells. On Fig. 2a, we observe an overexpression of the marker in mast cells, with a log2 FC of 6.48 for the median expression as compared to the median expression in all non-mast cell samples, as well as a log2 sFC of 5.94 of the median expression in mast cells as compared to the expression variance among all non-mast cell samples. Figure 2 b represents the ROC curve for this marker, showing an AUC of 0.997, thus providing sufficient sensitivity and specificity. After the automated screening, all retained transcripts were manually examined to remove transcripts that were also slightly expressed in other populations than the target populations, even though they fitted all three criteria (Additional file 1: Fig. S2). At this step, we found transcriptomic markers for 18 denominations (Additional file 1: Fig. S1b).

### Sub-signature selection

For some populations, a large number of transcriptomic markers were found (up to 41 for fibroblasts). A lower intra-signature correlation is likely to induce a loss of accuracy. To circumvent this potential issue, we selected a sub-signature for populations that had 8 or more markers, by choosing the highest inter-correlated set of markers (Fig. 2c and Additional file 1: Fig. S3). The final signatures are presented in Additional file 2: Table S1.

### Scoring metric

Given a cell population and its signature (i.e., the set of corresponding transcriptomic markers), the next step consisted in defining a metric, taking as input the expression value of this set of markers, and yielding as output a score of abundance of the cell population. To select a scoring metric, we performed tests on a dataset composed of in silico-simulated RNA mixtures (see the “Methods” section). Six scoring metrics were considered: arithmetic mean, geometric mean, harmonic mean, quadratic mean, energetic mean, and median. Each metric was tested on the mixtures by analyzing the correlations between the derived scores and the known proportions of all cell populations. The score for each scoring metric and each population are reported in Additional file 1: Fig. S4. All metrics were found to perform similarly. The median presents the advantage of being insensitive to outliers and was therefore chosen. At this point, we discarded the dermal dendritic cell signature, as the correlation between the scores and the mixture proportion for this population was below 0.75 (Additional file 1: Fig. S1b).

### Ex vivo validation

**Fig. 3:**
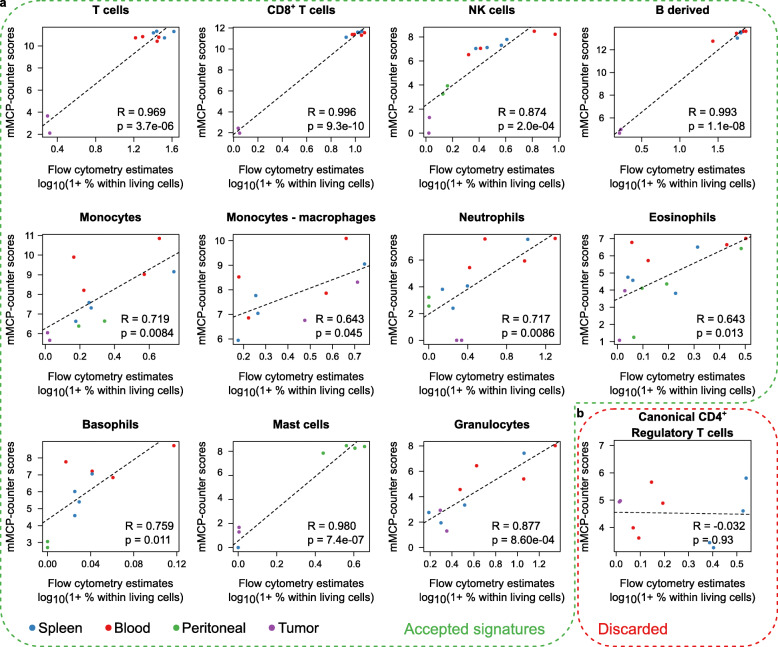
Validation of mMCP-counter on ex vivo data by comparison to flow cytometry data on *n* = 14 samples. **a** Correlation graphs between the flow cytometry estimates (logarithmic scale, expressed in percent of the total of living cells) and the mMCP-counter scores for populations for which the signature was accepted. Each graph corresponds to a different population. The dotted line shows the linear regression model. Correlations are estimated with the Pearson correlation. **b** Correlation graph for canonical CD4^+^ regulatory T cells presented as in **a**. Following this validation step, this signature was discarded

To validate our approach ex vivo, we analyzed 14 samples of the spleen (*n* = 4), peripheral blood (*n* = 4), peritoneum (*n* = 4), and TC1 tumors (*n* = 2) by flow cytometry and RNA-Seq and used the cytometry-estimated proportions of each cell type as reference (Additional file 1: Fig. S5). We applied mMCP-counter to the RNA-seq data and computed the correlation between the flow cytometry estimates (expressed in percentage within living cells) and the mMCP-counter scores for hematopoietic cell populations, pooling all samples regardless of the tissue of origin (Fig. 3). The signature for canonical CD4^+^ regulatory T cells failed this validation step (Fig. 3b and Additional file 1: Fig. S1b). However, for all other available populations (Fig. 3a), there was a good agreement between the mMCP-counter scores and the proportions obtained by flow cytometry, with correlation comprised between 0.629 (eosinophils) and 0.975 (CD8^+^ T cells).

### Comparison with other published methods

**Fig. 4:**
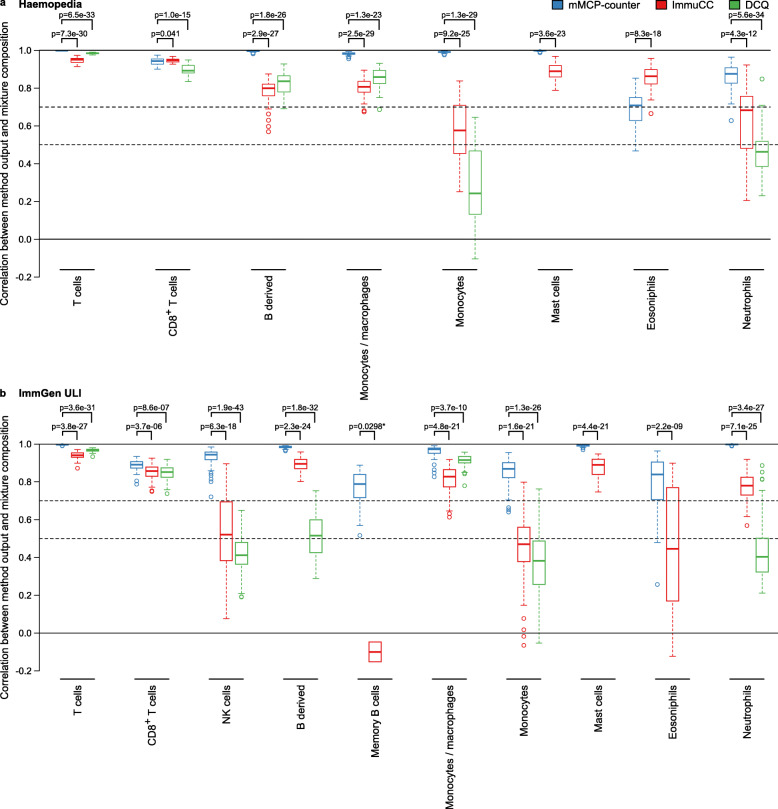
Comparison of the performance of mMCP-counter with other published methods. The three methods have been applied to 50 simulated RNA mixture sets, each comprising 50 randomized mixtures, generated from the Haemopedia (**a**) and ImmGen ULI datasets (**b**). This graph shows the Pearson correlation between the mixture compositions and the scores returned by the methods for each population on all mixture sets. Full lines indicate correlation equal to 0 and 1. Dashed lines indicate correlations equal to 0.7 and 0.5. An asterisk indicates that for memory B cells, there were only 2 datasets where ImmuCC returned non-all-zero scores and where its performance could therefore be assessed

Other methods have been previously reported to analyze the composition of heterogeneous samples in murine models [21, 22]. DCQ (Digital Cell Quantification) is an algorithm that, given gene expression data and prior knowledge on immune cell type transcriptomic profiles, returns the cell abundances for a wide variety of immune cells [21]; it was designed using RNA-Seq data. ImmuCC [22] is derived from the method proposed by CIBERSORT [23] and adapted by finding markers for murine populations; it was first designed for micro-arrays, but an updated version is adapted for RNA-Seq data [24]. We applied both methods on 50 sets of in silico RNA mixtures (each with 50 samples, see the “Methods” section) generated from the Haemopedia dataset [25] and 50 similar sets of 50 in silico mixtures generated from the ImmGen ultra-low-input RNA-seq (ImmGen ULI) dataset [26], another dataset composed of purified immune cells independent of the dataset used to generate the signatures, to assess each method’s performance on cell subtypes that were quantified by both mMCP-counter and another. On the Haemopedia dataset, mMCP-counter outperformed both ImmuCC and DCQ for T cells, B-derived cells, monocytes/macrophages, monocytes, mast cells, and neutrophils (all *p* values below 5e−12), performed similarly with ImmuCC and outperformed DCQ for CD8^+^ T cells, and was outperformed by ImmuCC only for eosinophils. On the ImmGen ULI dataset, which comprises more populations, including NK cells and memory B cells, on all considered populations, mMCP-counter outperformed both ImmuCC and DCQ (*p* < 4e−06 for all comparisons, with the exception of memory B cells, *p* = 0.0298 as ImmuCC computed non all-zero scores for the population for only 2 sets of mixtures). mMCP-counter also found to consistently perform well, with the median correlation between mixture compositions and scores above 0.7 for all considered populations in both datasets, whereas ImmuCC and DCQ performance greatly varied depending on the populations (Fig. 4).

We also estimated the spillover effect, that is the fact for a method to estimate the presence of other cell types in a setting with only one cell population present [5]. This is illustrated in Additional file 1: Fig. S6, for which the analysis was performed on the mean expression profile of CD8^+^ T cells, B-derived cells, NK cells, monocytes/macrophages, neutrophils, eosinophils, and mast cells from the Heamopedia (Additional file 1: Fig. S6a) and ImmGen ULI (Additional file 1: Fig. S6b) datasets. We found that mMCP-counter had a slight spillover where pure mast cells could have a non-zero score for B-derived cells, pure neutrophils a non-zero score for monocytes/macrophages and eosinophils, and monocytes/macrophages and eosiophils a non-zero score for each other. However, the overall noise ratio, measured as the ratio of off-target scores to total scores, was low (0.28 and 0.24 for Haemopedia and ImmGen ULI, respectively). ImmuCC had the lowest overall noise ratios of all three methods (0.07 and 0.19), but on the ImmGen ULI dataset, pure eosinophils are estimated as principally monocytes/macrophages. DCQ was found to have stronger spillover effects and overall noise ratios of 0.54 and 0.66.

### mMCP-counter discriminates tumor types and responders to immune checkpoint blockade

**Fig. 5:**
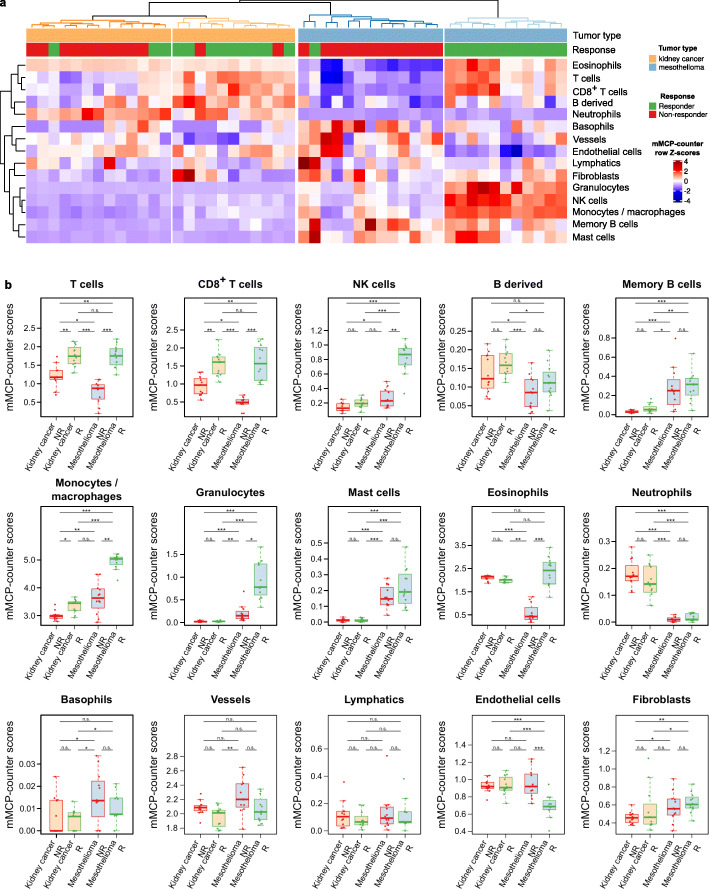
mMCP-counter discriminates between tumor types and between responders and non-responders to immune checkpoint blockade. **a** Heatmap showing that clustering of tumors on mMCP-counter scores accurately separates tumors based on the tumor type (*n* = 24 mesothelioma models, *n* = 24 kidney cancer models, first line) and the response to immune checkpoint blockade (second line, *n* = 12 responders and *n* = 12 non-responders for each cancer type). The heatmap illustrates row *Z*-scores for all included cell populations. **b** Detailed differences in mMCP-counter scores between responders and non-responders to ICB in both kidney cancer and mesothelioma models. Comparisons are computed using Kruskal-Wallis tests followed by post hoc Dunn test for pairwise comparisons, with Benjamini-Hochberg correction for multiple testing. *p < 0.05, **p < 0.01, ***p < 0.001, n.s. *p* ≥ 0.05

Immune checkpoint blockade (ICB) has become in the last decade a crucial treatment option for cancer patients. The response rate to such drugs strongly varies depending on the malignancy, and identifying patients likely to respond remains a challenge. Mouse pre-clinical models greatly help to identify markers of response that are potentially useful in human clinical trials. We therefore applied mMCP-counter to pre-treatment samples of mouse models of kidney cancer and mesothelioma that have been treated with a combination of CTLA-4 and PD-L1 blockade [27]. We could therefore investigate whether mMCP counter could detect differences in the tumor micro-environment (TME) composition between cancer types and between tumors responding or not to ICB. An unsupervised analysis (Fig. 5a) revealed that the TME, as analyzed by mMCP-counter, principally discriminates malignancies based on the tumor type. Within each tumor type, the unsupervised clustering on the mMCP-counter scores allows to discriminate between two groups associated with response to ICB, suggesting that the TME composition is tightly associated with response to ICB.

In more details, we also analyzed the association between the scores for each population and response, in both models (Fig. 5b). This revealed associations with responses that are found in both kidney cancer and mesothelioma models. Indeed, the two models showed that responsive tumors had an increased infiltration by T cells, CD8^+^ T cells, and monocytes/macrophages as compared to tumors that resisted the ICB treatment. However, other TME differences between responders and non-responders appear to be cancer type-specific. Thus, in mesothelioma, responders exhibited more NK cells, granulocytes, and eosinophils, and less endothelial cells than non-responders, while these associations were not found in kidney cancer models. Moreover, some populations were particularly differentially present in the two tumor types, including NK cells, B-derived cells, memory B cells, monocytes/macrophages, granulocytes, mast cells, neutrophils, basophils, and fibroblasts.

### mMCP-counter identifies immune and stromal correlates of early Alzheimer’s disease onset

**Fig. 6:**
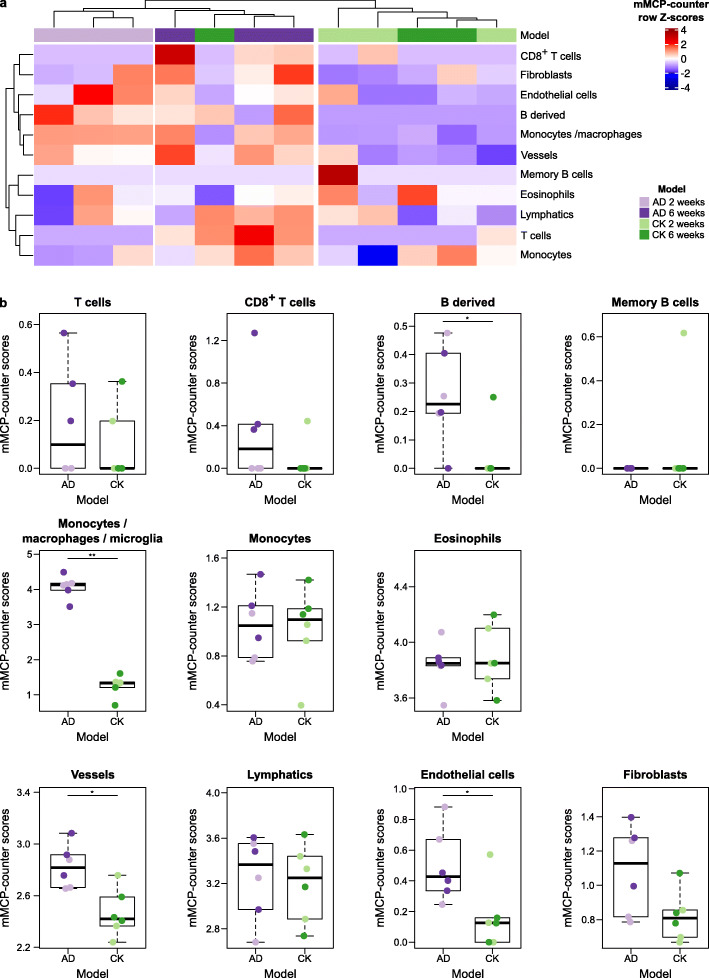
mMCP-counter discriminates between control CK mice and Alzheimer’s disease brain tissues. **a** Heatmap showing that clustering of samples on mMCP-counter scores accurately separates hippocampus samples from control CK samples and induced Alzheimer’s disease (AD) at different time points (*n* = 6 AD and *n* = 6 CK). The heatmap illustrates row *Z*-scores for all included cell populations. **b** Detailed differences in mMCP-counter scores between CK and induced AD hippocampus samples. The color code of the individual data points refers to the legend of panel **a**. Comparisons are computed using Mann-Whitney tests. *p < 0.05, **p < 0.01

Alzheimer’s disease (AD) has been modeled by a bitransgenic mouse model called CK-p25 which can overexpress p25 when induced through the calcium/calmodulin-dependent protein kinase II (CK) promoter, as compared to CK control mice [28]. p25 triggers an aberrant activation of cyclin-dependent kinase 5, which in turn increases phosphorylation of pathological substrates including tau. We obtained RNA-seq data from the hippocampus of CK-p25 mice, thereafter labeled as AD mice, at 2 or 6 weeks into neurodegeneration, as well as similar data from control CK mice [29]. Using mMCP-counter, we observed that the immune and stromal composition of the samples neatly segregated mice with AD from CK mice (Fig. 6a), suggesting that AD impacts the hippocampus’ immune infiltration and vascularization.

In detail, we notably observed that AD mice hippocampus had a higher infiltration by B-derived cells, but not memory B cells, more macrophages (since they have an increased score for monocytes/macrophages, but not monocytes), and more endothelial vessels (Fig. 6b). The increase in macrophages is likely due to microglia, the resident macrophages of the central nervous system. Indeed, using single-cell RNA-sequencing data from the *Tabula Muris* project [14], we noticed a strong expression of the monocytes/macrophages signature by microglial cells in the brain (Additional file 1: Fig. S7). No significant alterations were found for T cells, eosinophils, lymphatics, and fibroblasts. We noted that 6-week AD mice showed an increased presence of CD8^+^ T cells in the hippocampus, although the limited number of mice did not allow to reach significance (*p* = 0.01).

## Discussion

Here we introduced mMCP-counter, a method to quantify immune and stromal cell populations in heterogeneous murine samples. mMCP-counter is based on the identification of highly specific transcriptomic signatures for each of the cell populations considered. We found robust signatures for a total of 16 populations: 12 immune populations (T cells, CD8^+^ T cells, NK cells, B-derived cells, memory B cells, monocytes/macrophages, monocytes, granulocytes, mast cells, eosinophils, neutrophils, and basophils) and 4 stromal populations (vessels, lymphatics, endothelial cells, and fibroblasts).

We validated mMCP-counter method by comparing the scores with flow cytometry estimates on blood, spleen, peritoneal, and tumor samples and demonstrate a strong correlation between both methodologies. For several populations, a very strong correlation was found, in part due to the fact that we included samples with both very low and very high content in these populations. While this limits the possibility to demonstrate a proper linearity between the mMCP-counter scores and the flow cytometry estimates, it shows that no outlier was found, and that mMCP-counter consistently returned extremely low or high scores for samples with low or high content, respectively.

We also showed that mMCP-counter allows a significant improvement over the existing methods by comparing its performance to previously developed methods on large simulated mixture datasets from two independent datasets.

Applied to mouse models of kidney cancer and mesothelioma treated by combination of immune checkpoint blockade therapies, mMCP-counter allows to decipher the differences of tumor microenvironment composition between both tumor types and between responders and non-responders to immune checkpoint blockade. Alongside known associations between TME composition and response to ICB, such as T cells and CD8^+^ T cells, mMCP-counter revealed that tumors responsive to ICB had a higher infiltration by monocytes and/or macrophages. Moreover, malignancy-specific differences between responders and non-responders could be observed, that were not previously reported. Finally, there were strong differences in the overall composition of the TME between mesothelioma and kidney cancer models. Due to the rapidly increasing, almost impossible to handle, number of agents tested in immunotherapy clinical trials [30, 31], it is of paramount importance to test them in pre-clinical models with a method that robustly and sensitively quantifies the TME composition. Altogether, mMCP-counter may help find the rationale for potential cancer-specific combination strategies and drive more efficient personalized cancer medicine.

To assess the applicability of mMCP-counter beyond the field of cancer, we analyzed a model of Alzheimer’s disease. Thus, we applied mMCP-counter to hippocampal transcriptomics data from a murine model of Alzheimer’s disease, comparing mice with induced AD with controls. The most striking difference was an increased expression of the monocyte/macrophage signature in AD mice. This may be explained by the detection by mMCP-counter of microglia, which have been shown to be a prominent marker of AD [32]. Conversely, the role of B cells is disputed and looked as inessential in AD [32]. Here, we also noticed an increase in B lineage cells. Finally, we also noted an increased presence of endothelial vessels in AD mice, in line with reports of angiogenesis in AD [33]. mMCP-counter could help further analyze the immune and stromal impacts of AD and other neurodegenerative syndromes. Although it did not reach significance, a trend towards an increase in the presence of CD8^+^ T cells in the hippocampus of AD mice between weeks 2 and 6 was observed. This concords with recent observation of presence of CD8^+^ T cells in the hippocampi of AD human patients [34].

mMCP-counter is fast and memory-efficient to compute the scores. The abundance scores it provides are shown to be linearly related to the known abundances of the related cell populations in validation data. These scores can thus safely be compared across the samples of a given series, as illustrated here in two examples. mMCP-counter scores are given in population-dependent arbitrary units. As such, the intra-sample ratio of the scores of two distinct cell populations is not an accurate estimate of the actual intra-sample ratio of these two populations, as reported for MCP-counter [35]. However, such a ratio could still be compared across samples within a series, as by construction, it would also be linearly correlated to true ratios. This is a major difference with some methods, including CIBERSORT-based ImmuCC, which instead enable intra-sample comparison but do not allow inter-sample comparisons as it returns the proportions of immune cells within the overall immune infiltrate, not within the full sample [5, 35]. Therefore, samples with similar relative composition of the immune infiltrate, but one highly infiltrated and the other lowly infiltrated, would have similar ImmuCC outputs. CIBERSORT, but not ImmuCC, now offers an absolute version of their tool which removes this obstacle [36]. Although mMCP-counter allows a robust quantification of 16 immune and stromal cell populations, the functional orientation cannot be assessed using mMCP-counter. In particular, this tool does not allow precise quantification of immunosuppressive populations such as regulatory T cells or macrophage polarization.

For human samples, a large number of methods are available and are part of a global set of methods to study cancer immunity [37]. However, they differ in their performance, and signatures appear to be the most critical aspect of such approaches [38]. The robust and stringent definition of signatures of MCP-counter allows it to be among the best performing ones [5]. However, only few of these methods were available for murine models to this day. Here, we have kept the same methodology to define gene signatures that are highly specific for the considered cell populations, which could explain that mMCP-counter outperforms the other approaches. Indeed, to build mMCP-counter, we chose to use very stringent definitions of specific transcriptomic markers. This allows a precise estimation of all measured populations, but it is at the expense of the number of populations that can be accounted for. For instance, mMCP-counter does not include quantification of CD4^+^ T cells. Although this is a crucial cell population in many settings, we would have had to diminish the signature quality cut-offs, at the expense of a lower accuracy of mMCP-counter. Other methods, such as DCQ, estimate changes in more than 70 immune populations, where we only quantify 12 plus 4 stromal populations. mMCP-counter therefore cannot estimate all precise functional orientations but outperforms DCQ for the populations where it applies. Nevertheless, robust signatures for memory B cells were identified here. Similarly, there is a growing interest in the heterogeneity of cancer-associated fibroblasts [39, 40]. To include more details as to fibroblasts subtypes in mMCP-counter would have required far more detailed data on sorted fibroblasts subtypes than what is currently available.

## Conclusions

Although many different methods are currently available to estimate the immune and stromal composition of heterogeneous human tissues, only a few such methods have been reported for murine samples. In the present study, we have introduced and validated mMCP-counter, a method that allows a precise estimation of the abundance of 12 immune and 4 stromal populations in murine tissues from transcriptomic data.

mMCP-counter can provide extremely useful information in cancer murine models. A major asset of mMCP-counter, compared to previously reported methods, is that it allows to simultaneously study immune and stromal cell populations. The clinical relevance of the tumor microenvironment composition is not only documented for immune cells [3], but also for stromal cells: blood vessels and angiogenesis are key players in cancer development and metastasis [41], lymphatic vessels are associated with metastasis [42], and the impact of fibroblasts raises a growing interest [43].

Beyond the field of cancer, mMCP-counter may also have broad applications in murine models of diseases in which immunity, inflammation, or angiogenesis play crucial roles. In particular, it can be applied to models of neurodegenerative syndromes such as Alzheimer’s disease, well-known auto-immune [44] and inflammatory [45, 46] diseases, but also atherosclerosis [47].

## Supplementary information

**Additional file 1.** PDF (.pdf) file. Supplementary figures S1 to S7.

**Additional file 2.** Excel spreadsheet (.xlsx). Supplementary Table S1. Signatures used by mMCP-counter. The signatures are indicated in the following format: Affymetrix Mouse Gene 1.0 ST Array probe ID, HUGO gene symbol and ENSEMBL gene ID.

## Data Availability

mMCP-counter can be accessed as an R package (https://github.com/cit-bioinfo/mMCP-counter) [9]. The RNA-seq data from murine spleen, peritoneum, peripheral blood, and tumors are available from ArrayExpress (accession code E-MTAB-9271) [48].
