# Supplementary material for: The murine Microenvironment Cell Population counter method to estimate abundance of tissue-infiltrating immune and stromal cell populations in murine samples using gene expression
Source: Genome Med. 2020 Oct 6;12:86. doi: 10.1186/s13073-020-00783-w (PMC7541325; doi:10.1186/s13073-020-00783-w)
Supplement: Supplementary file 1 — Additional file 1. PDF (.pdf) file. Supplementary figures S1 to S7. [file 13073_2020_783_MOESM1_ESM.pdf]

a

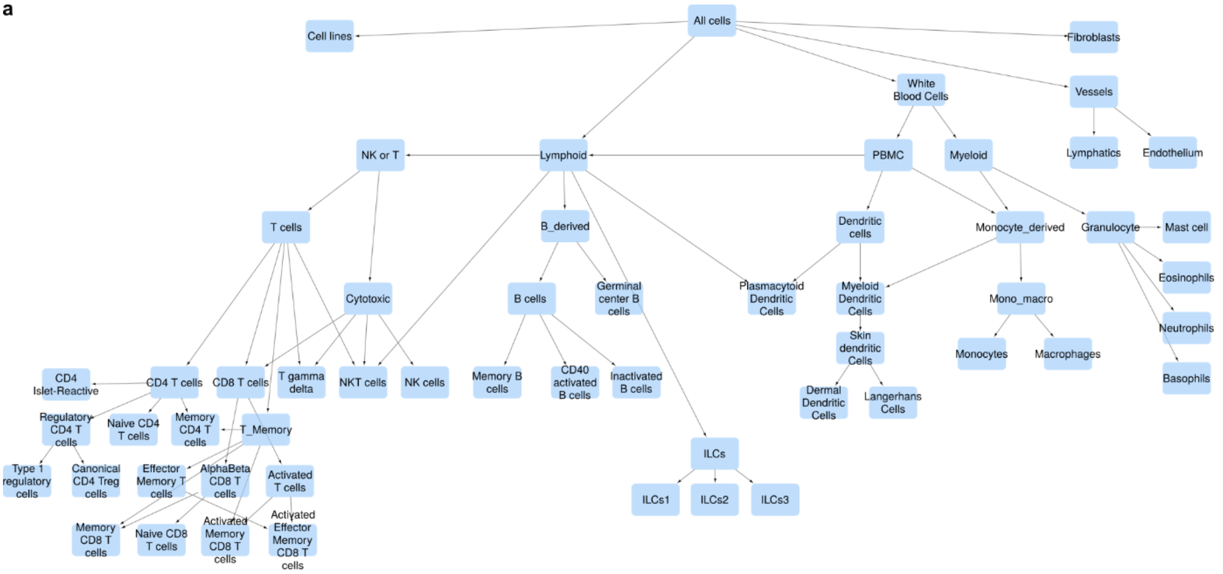

b

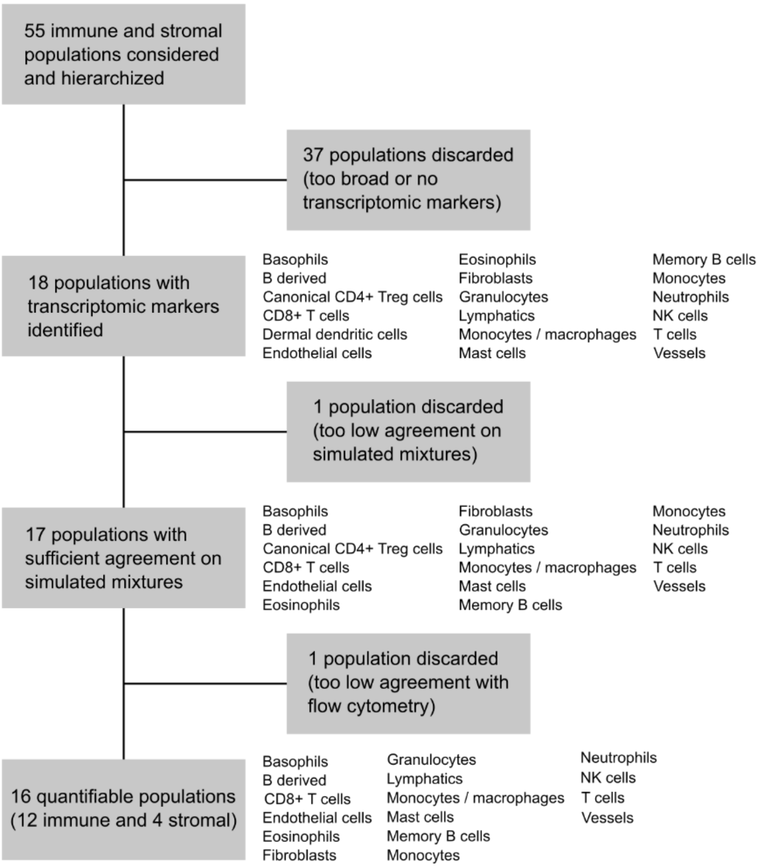

Fig S1: Cell populations and signatures

a Cell populations hierarchy used in the present study. **b** Counts of the number of signatures identified and discarded during the various validation steps.

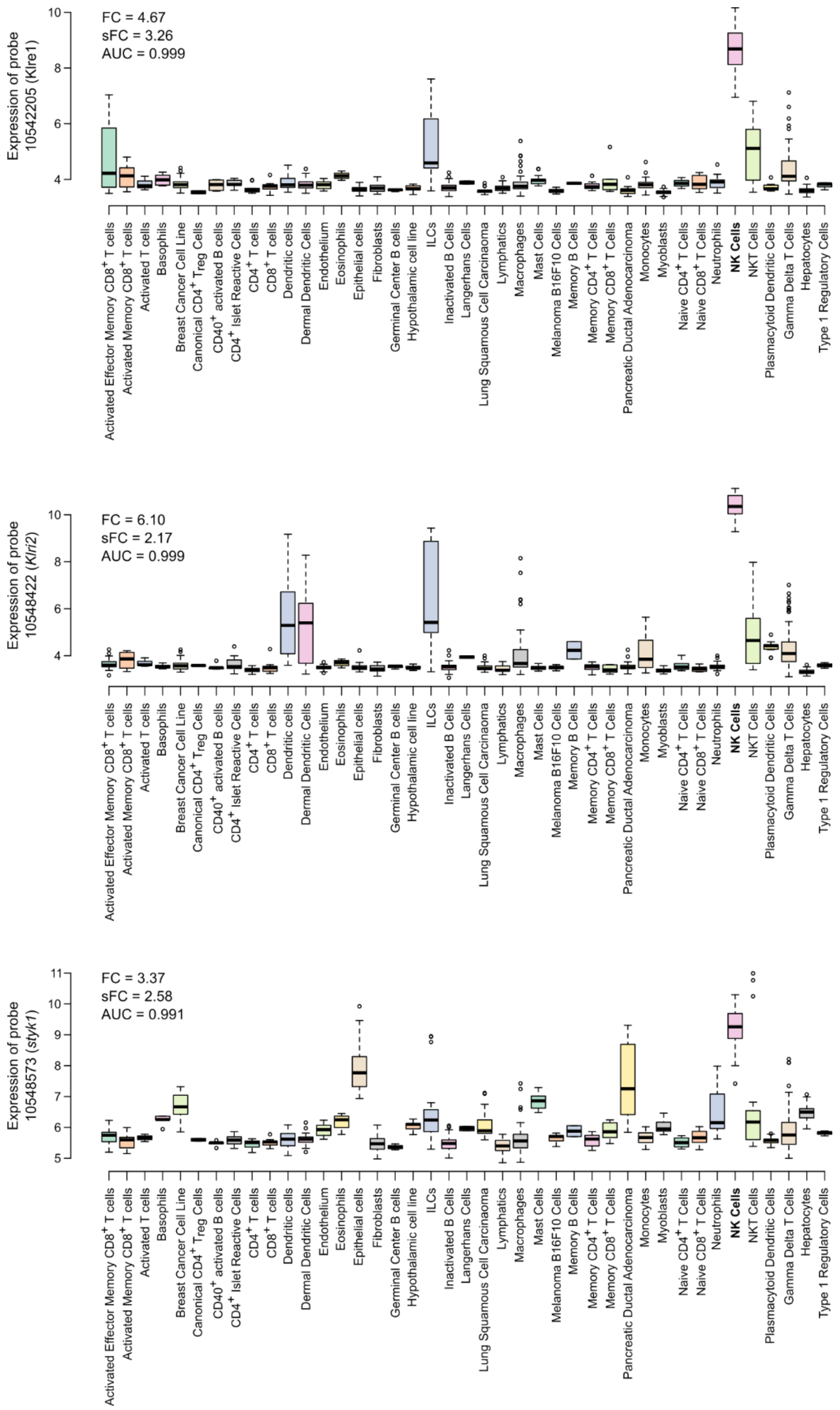

Fig S2: Examples of discarded transcriptomic markers

The three markers presented here satisfied the criteria to be considered as transcriptomic markers for NK cells, but they were discarded during manual curation due to insufficient specificity.

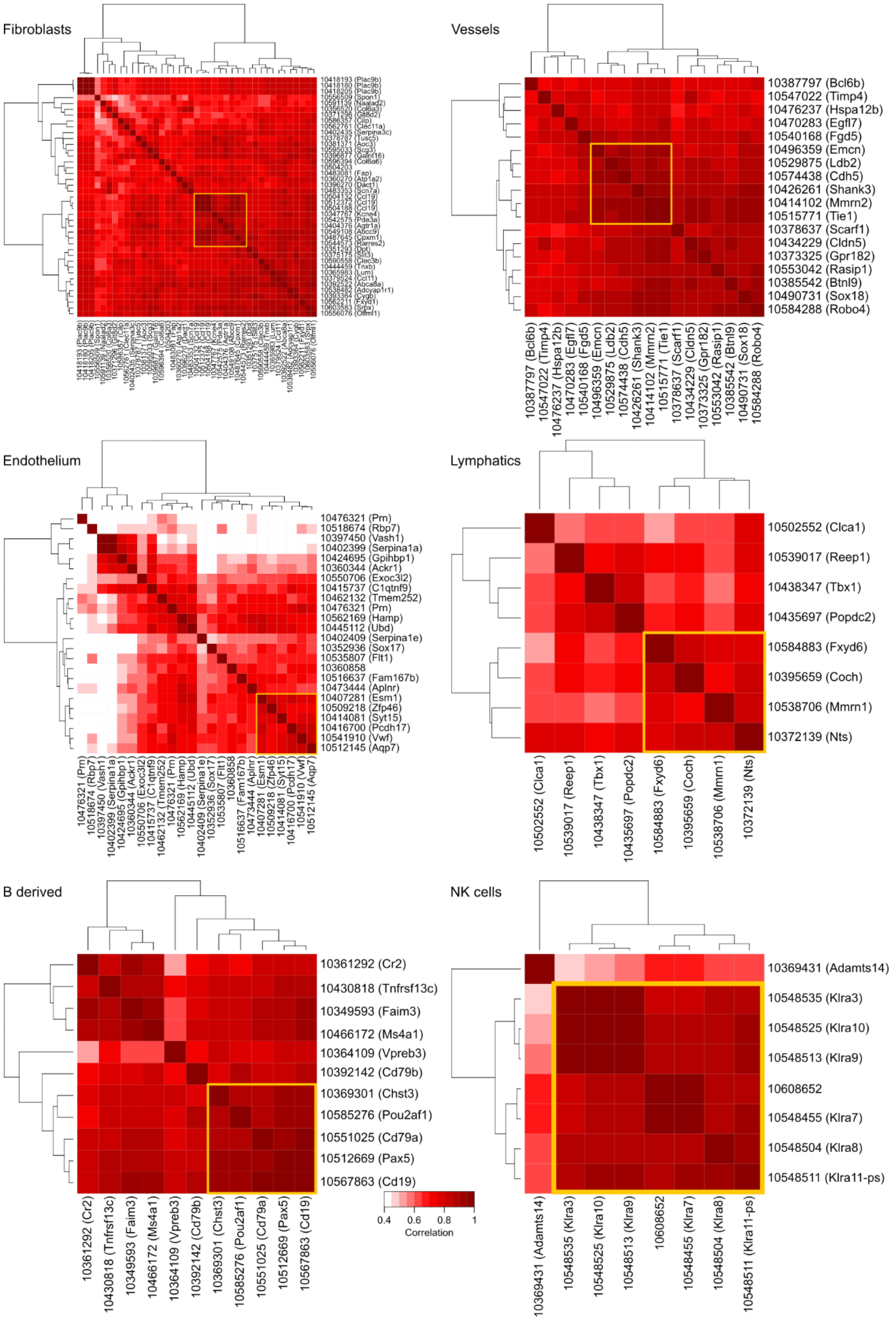

Fig S3: Selection of signatures for populations with numerous markers.

Each heatmap is associated with a population that had 8 or more transcriptomic markers. The yellow rectangle indicates the chosen signature.

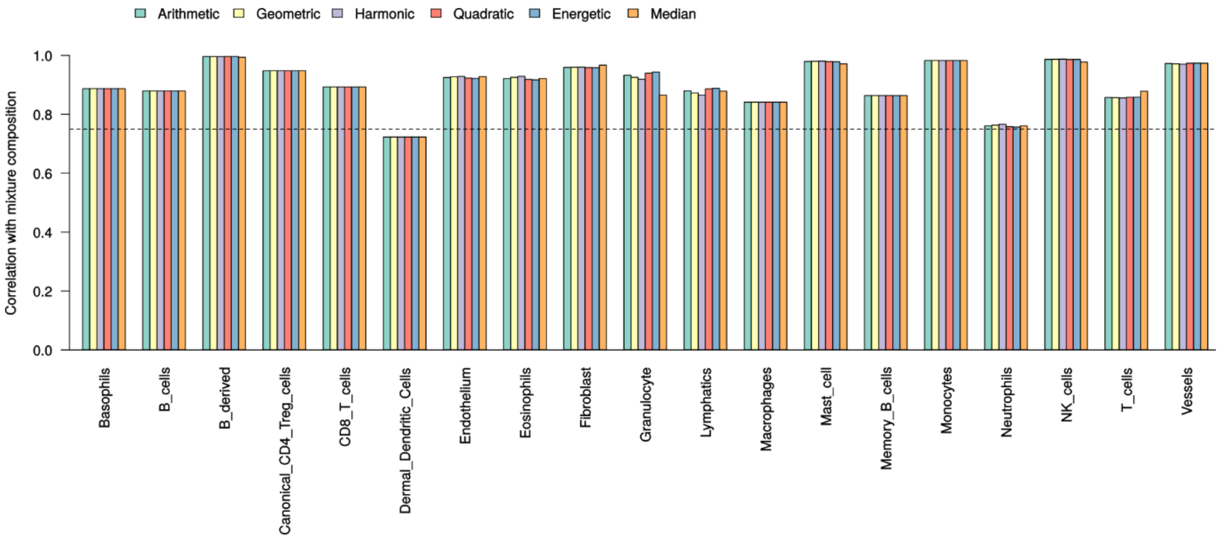

Fig S4: Comparison of different scoring methods.

This figure relates to in silico simulated RNA mixtures. For each cell population, the correlation between the mixtures' compositions and the expression the of the signature was compared for various scoring methods.

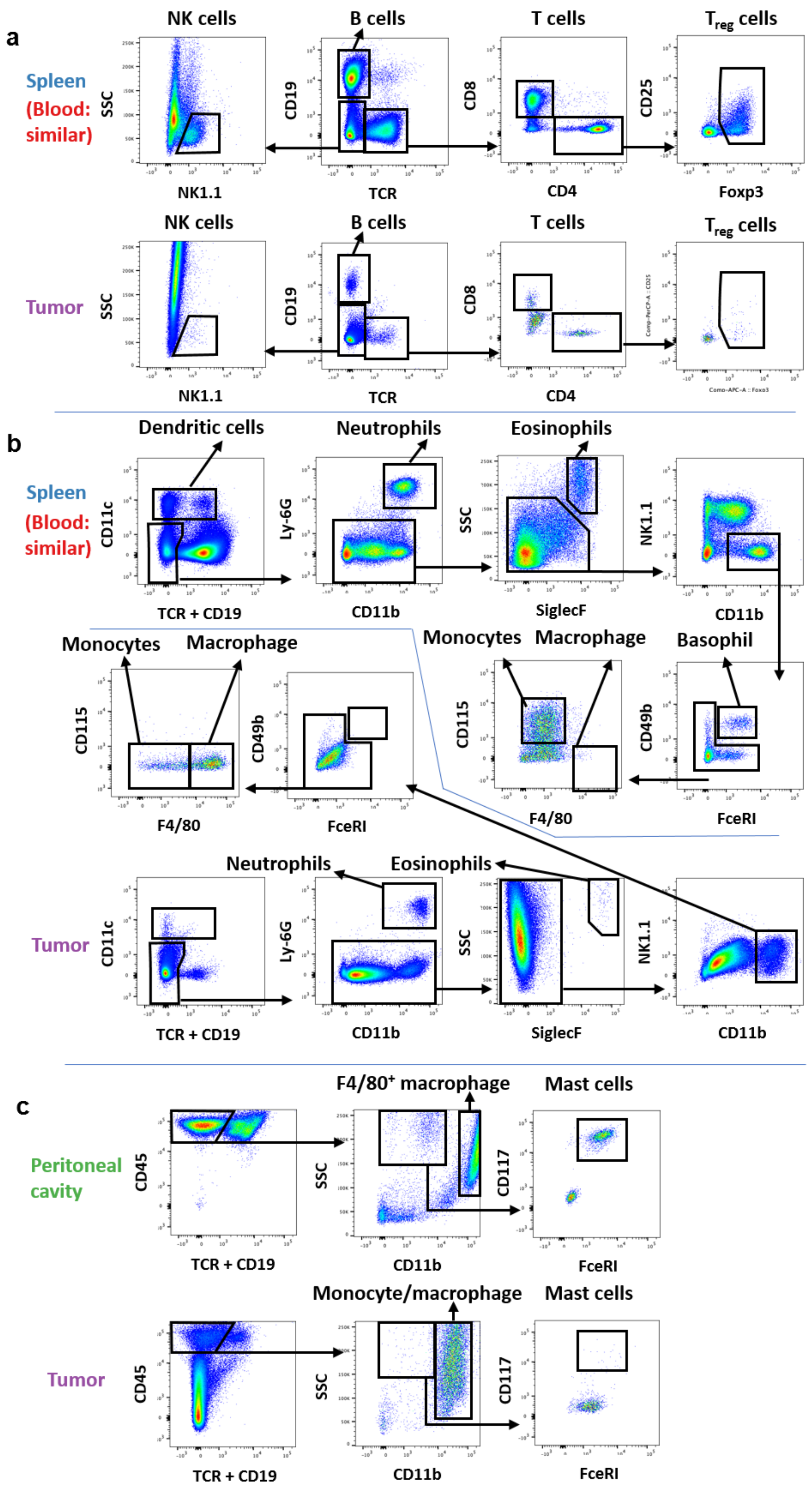
a
NK cells
B cells
T cells
T_{reg} cells
Spleen
(Blood: similar)
SSC
NK1.1
CD19
TCR
CD8
CD4
CD25
Foxp3
Tumor
b
Dendritic cells
Neutrophils
Eosinophils
CD11c
TCR + CD19
Ly-6G
CD11b
SiglecF
NK1.1
Monocytes
Macrophage
Basophil
CD115
F4/80
CD49b
FceRI
c
F4/80+ macrophage
Mast cells
Peritoneal cavity
CD45
CD117
Monocyte/macrophage

Fig S5: Representative gating strategy of flow cytometry analyses for **a.** T cells, B cells and NK cells; **b.** dendritic cells, granulocytes, monocytes and macrophages; and **c.** mast cells and peritoneal macrophages. Blood samples were not shown due to high similarity of the gating strategy to splenocytes

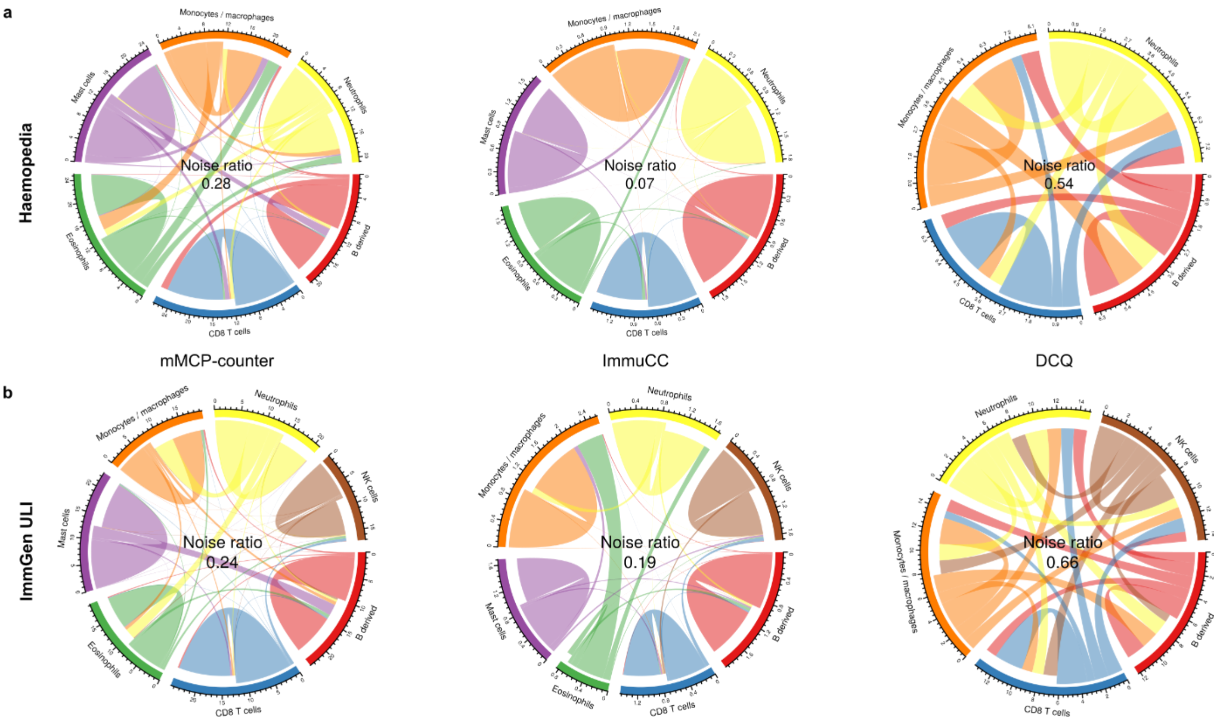

Fig S6: Spillover analysis and noise ratio for the three methods on the Haemopedia (**a**) and ImmGen ULI (**b**) datasets. The circos plots indicate spillover experiment outputs. Each color corresponds to a cell population, and the estimates for pure profiles are represented by the arcs inside the plot. These arcs are color-coded according to the true cell type that was given as an input to the method, and their size represents the weight given to other cell types by the methods.

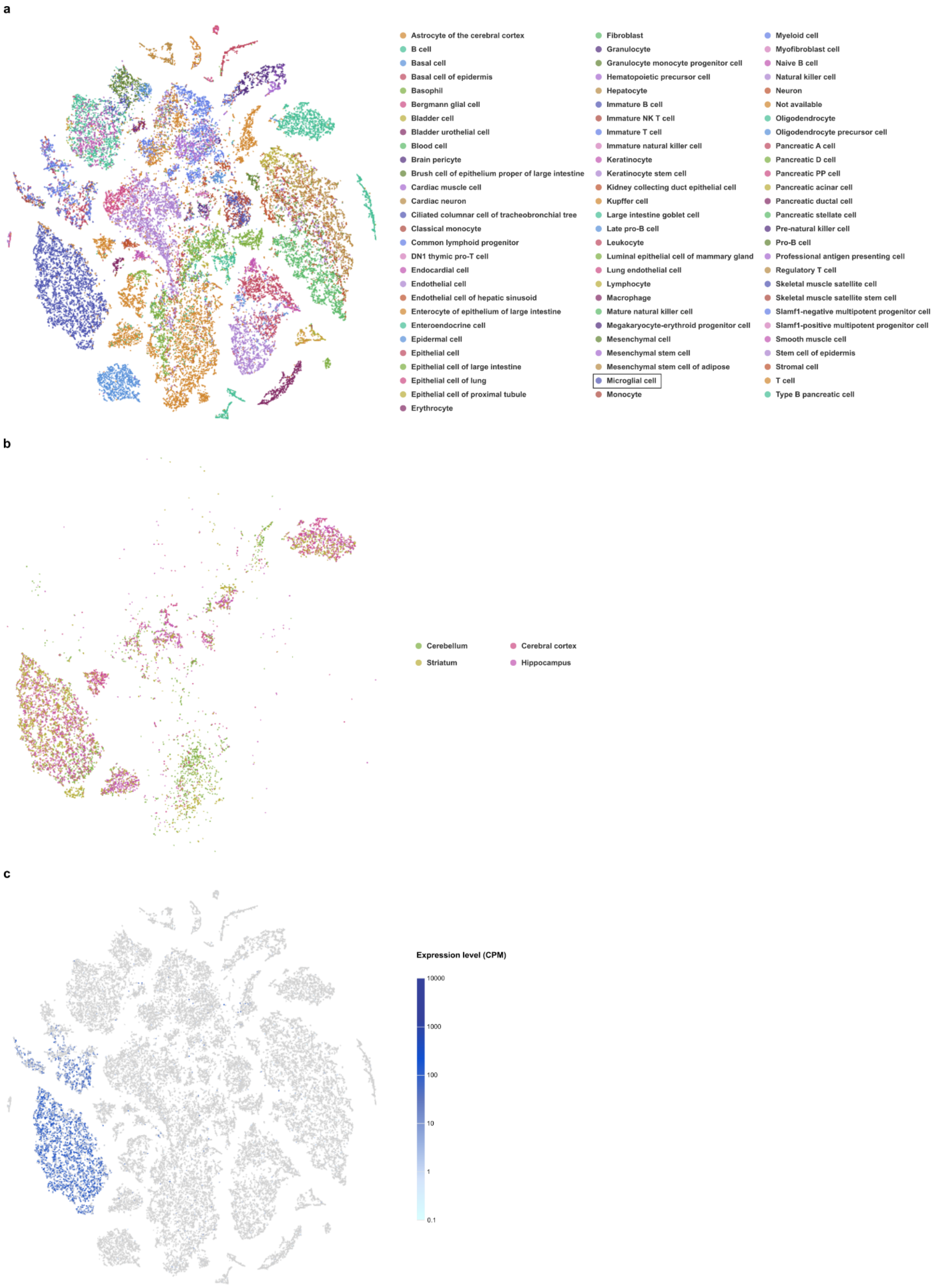

Fig S7: The mMCP-counter signature for monocytes / macrophages is also expressed in microglial cells in the brain

This figure was generated using the Elixir platform from single-cell RNA-seq data from the Tabula Muris consortium.

a. t-SNE representation of the full dataset, colored by inferred cell type. This notably shows a cluster of microglial cells, dark blue, left of the plot.
b. Position of brain cells in the same t-SNE plot, colored by region of origin.
c. Expression level of the mMCP-counter signature for monocytes/macrophages, showing a strong expression in several cell clusters, including the microglial cell cluster.
